# A Naturally Occurring Isoform Inhibits Parathyroid Hormone Receptor Trafficking and Signaling

**DOI:** 10.1002/jbmr.167

**Published:** 2010-06-24

**Authors:** Verónica Alonso, Juan A Ardura, Bin Wang, W Bruce Sneddon, Peter A Friedman

**Affiliations:** 1 Laboratory for G Protein–Coupled Receptor Biology, Department of Pharmacology and Chemical Biology, University of Pittsburgh School of MedicinePittsburgh, PA, USA; 2 Department of Biological Sciences, Duquesne UniversityPittsburgh, PA, USA

**Keywords:** PTH RECEPTOR, ISOFORM, DOMINANT-NEGATIVE, ALTERNATIVE SPLICING, G PROTEIN–COUPLED RECEPTORS, MEMBRANE TRAFFICKING, MAP KINASE, ADENYLYL CYCLASE

## Abstract

Parathyroid hormone (PTH) regulates calcium homeostasis and bone remodeling through its cognitive receptor (PTHR). We describe here a PTHR isoform harboring an in-frame 42-bp deletion of exon 14 (Δe14-PTHR) that encodes transmembrane domain 7. Δe14-PTHR was detected in human kidney and buccal epithelial cells. We characterized its topology, cellular localization, and signaling, as well as its interactions with PTHR. The C-terminus of the Δe14-PTHR is extracellular, and cell surface expression is strikingly reduced compared with the PTHR. Δe14-PTHR displayed impaired trafficking and accumulated in endoplasmic reticulum. Signaling and activation of cAMP and ERK by Δe14-PTHR was decreased significantly compared with PTHR. Δe14-PTHR acts as a functional dominant-negative by suppressing the action of PTHR. Cells cotransfected with both receptors exhibit markedly reduced PTHR cell membrane expression, colocalization with Δe14-PTHR in endoplasmic reticulum, and diminished cAMP activation and ERK phosphorylation in response to challenge with PTH. Δe14-PTHR forms heterodimers with PTHR, which may account for cytoplasmic retention of PTHR in the presence of Δe14-PTHR. Analysis of the PTHR heteronuclear RNA suggests that base-pair complementarity in introns surrounding exon 14 causes exon skipping and accounts for generation of the Δe14-PTHR isoform. Thus Δe14-PTHR is a poorly functional receptor that acts as a dominant-negative of PTHR trafficking and signaling and may contribute to PTH resistance. © 2011 American Society for Bone and Mineral Research.

## Introduction

Type I parathyroid hormone (PTH) and PTH-related peptide receptor (PTHR) belong to family B, subfamily 1, of G protein–coupled receptors (GPCRs). Other members include receptors for secretin, vasoactive intestinal peptide, growth hormone–releasing hormone, glucagon, glucagon-like peptide, pituitary adenylyl cyclase–activating peptide, corticotropin-releasing hormone, and calcitonin (CTR).(1) The PTHR is expressed predominantly in kidney and bone, where it mediates PTH actions on calcium and phosphate homeostasis and bone turnover, respectively.(2)

In humans, the *PTHR* gene contains 15 exons* coding a 593-amino-acid, 7-transmembrane-domain (TMD) receptor.(3,4) Family B1 GPCRs are characterized by an exon-intron organization that permits alternative splicing of specific critical domains that have been shown in some instances to alter the function of the resulting isoform.(5) Some of these family B isoforms are characterized by the deletion of regions encoding the seventh TMD (TMD7).(5–8) The biologic role of these isoforms is largely unexplored, but studies with corticotropin-releasing hormone receptor (CRHR) variants suggest that they could be cellular response modulators affecting CRHR signaling.(6) Several PTHR isoforms, or transcripts consistent with receptor isoforms, have been described.(9–11) It has been suggested that presumptive nonfunctional PTHR isoforms could be the source of pathologies associated with PTH dysfunction, including some cases of pseudohypoparathyroidism type Ib (PHPIb).(12) Analysis of the exon coding structure and promoter regions of the *PTHR* gene or its mRNA, however, failed to disclose mutations.(13–16) The biologic behavior and functional consequence of alternatively spliced PTHR forms on signaling and trafficking and their effects on PTHR action are unknown. We now show the existence of a PTHR isoform lacking TMD7, which is encoded by exon 14 (Δe14-PTHR), in human renal epithelial cells. We characterized Δe14-PTHR and its actions as a modulator of PTHR. Δe14-PTHR expression is primarily cytoplasmic, where it interacts with the PTHR in endoplasmic reticulum, thereby reducing delivery of the wild-type receptor to the cell membrane and simultaneously promoting *PTHR* downregulation. Nonetheless, some Δe14-PTHR is expressed at the plasma membrane, but the absence of TMD7 results in extracellular localization of C-terminal receptor tail. Signaling via cAMP formation and p44/42 MAP kinase [extracellular signal-regulated kinase (ERK)] phosphorylation were decreased in response to PTH. Δe14-PTHR also decreases cAMP and ERK responses when coexpressed with the fully active PTHR. We conclude that Δe14-PTHR acts as a dominant-negative of PTHR and causes PTH resistance.

The exon nomenclature and numbering for the *PTHR* are confusing. The literature and PubMed give 14 to 16 exons. Exon 1 is the first that includes the start site of transcription and, as such, is not defined by the start site of translation or the start site of the mature protein. As with most genes, the data on the true exon 1 (where transcription starts) is incomplete. Evidence suggests that there are multiple forms of exon 1 that are tissue-specific. There is at least 1 exon before the exon encoding the signal sequence, which is exon 2. Based on this consideration, there are tentatively 15 exons in the human, mouse, and rat *PTHR* genes. Additionally, a preliminary description of the *PTHR* lacking helix 7 referred to it as Δe14-PTHR.(12) For these reasons, we follow the same numbering.

## Materials and Methods

### Reagents

Polyclonal and monoclonal HA.11 and monoclonal antihistidine (His) antibodies were obtained from Covance (Berkeley, CA, USA). Monoclonal anti-Flag antibody was purchased from Sigma (St Louis, MO, USA). The phosphorylated ERK1/2 and total ERK antibodies were obtained from Cell Signaling Technology (Danvers, MA, USA). Polyclonal anti-lysosome-associated membrane protein 2 (anti-LAMP-2) was obtained from Anaspec (San Jose, CA, USA). Secondary antibodies Alexa-Fluor 488, Alexa-Fluor 546, Alexa-Fluor 680, zeocin, blasticidin, and geneticin were purchased from Invitrogen (Carlsbad, CA, USA). The endoplasmic reticulum–selective, cell-permeant dye ER-Tracker Red (BODIPY TR Glibenclamide) and the nuclear counterstain 4',6-diamidino-2-phenylindole (DAPI) were purchased from Invitrogen. Horseradish peroxidase (HRP)–conjugated goat antirabbit secondary antibody was from Pierce (Rockford, IL, USA), and HRP-conjugated sheep antimouse antibody was from GE Healthcare (Piscataway, NJ, USA). Protease inhibitor mixture set I was from Calbiochem (San Diego, CA, USA). Human PTH(1–34) and PTH(7–34) were obtained from Bachem (Torrance, CA, USA). All other reagents were from Sigma.

### Cell culture

Renal proximal tubule cells were isolated from the urine of normal subjects as described previously.(17) These cells exhibit a phenotype that includes expression of γ-glutamyl transpeptidase, a characteristic brush-border enzyme and PTH-stimulated cAMP.(18) Briefly, urine samples were centrifuged for 15 minutes at 1500*g* at 4°C, and pellets were washed twice with phosphate-buffered saline (PBS). Cell RNA was isolated using guanidinium thiocyanate–phenol–chloroform extraction (TRIZOL, Invitrogen) according to the manufacturer's instructions. Buccal epithelials cells were harvested with a cotton swab, and RNA was isolated as described previously. CHO-N10 cells, a subline of Chinese hamster ovary developed in our lab,(19) were cultured in Ham's F-12 medium supplemented with 10% fetal bovine serum (FBS), 100 units/mL of penicillin, 100 µg/mL of streptomycin, and 10 µg/mL of blasticidin. HEK-293 cells were cultured in Dulbecco's modified Eagle's medium (DMEM) supplemented with 10% FBS, 100 units/mL of penicillin, and 100 µg/mL of streptomycin. Also, 1.5% G418 was added to the latter medium used for HEK-293 cells constitutively expressing the GFP-PTHR (HEK-293R).(19) Immortalized proximal tubule epithelial HK-2 and HKC-8 cells from normal adult human kidney(18) were cultured in DMEM/F-12 50:50 medium supplemented with 10% FBS, 100 units/mL of penicillin, and 100 µg/mL of streptomycin. Cells were maintained at 37°C in a humidified atmosphere of 5% CO_2_

### Plasmid constructions

pcDNA3.1+-HA-PTHR, pcDNA3.1+-Flag-PTHR, pcDNA3.1+-HA-Δe14-PTHR, pcDNA3.1+-Flag-Δe14-PTHR, pBudCE4.1+-Flag-PTHR-His, and pBudCE4.1+-HA-Δe14-PTHR–hemagglutinin (HA)–tagged human PTHR in pcDNA3.1 were constructed as described previously.(20)

### pcDNA3.1+-Flag-PTHR, pcDNA3.1+-HA-Δe14-PTHR, and pcDNA3.1+-Flag-Δe14-PTHR

Flag-tagged PTHR was generated by converting the sequence DKEAPTGS (residues 94 to 101) in exon E2 to DYKDDDDK of Flag epitope.(21) pcDNA3.1(+)-HA-Δe14-PTHR was engineered by using polymerase chain reaction (PCR) overlapping extension for two-fragment assembly.(21) Briefly, a 1.4-kb fragment from amino acids 1 to 451, with incorporation of a *Hin*dIII restriction at the 5' site, was amplified by PCR using pcDNA3.1(+)-HA-PTHR as a template.(19) A second fragment of 0.4-kb product from amino acid 466 to the end of PTHR with incorporation of a 15-bp extension at the 5' site, which overlapped with the 3' site of the first fragment, and *Eco*RI at the 3' site was amplified by PCR using the same template as for the first fragment synthesis. The second PCR was performed using the preceding two fragments as templates. HA-Δe14-PTHR was subcloned into pcDNA3.1(+). pcDNA3.1(+)-Flag-Δe14-PTHR was engineered as earlier except that pcDNA3.1(+)-Flag-PTHR served as the PCR template. The accuracy of these constructs was confirmed by sequencing (ABI PRISM 377, Applied Biosystems, Foster City, CA, USA).

pBudCE4.1+-Flag-PTHR-His and HA-Δe14-PTHR-His were obtained in the following manner: Flag-PTHR and HA-Δe14-PTHR were amplified using the forward primer with *Not*I restriction site (AGAAGAAGAAAGCGGCCGCATGGGGACCGCCCGGATC), and the reverse primer with *Bst*BI restriction site (CGGAGGAGAATTTCGAACATGACTGTCTCCCACTC). Purified PCR fragments were cut by *Not*I and *Bst*BI and subcloned into the pBudCE4.1 before a polyhistidine-expressing region.

### Transient transfection

Cells were grown to 50% to 60% confluence and transfected, as indicated with 1 µg of DNA per well in 6-well plates with HA-PTHR, Flag-PTHR, HA-Δe14-PTHR, Flag-Δe14-PTHR, and EPAC, Rab 5, Rab 7, Rab 11, and Arf 1(22) (kindly provided by Dr J-P Vilardaga) using FuGENE 6 (Roche, Indianapolis, IN, USA) according to the manufacturer's protocol. Experiments involving transfection of PTHR isoforms, Rabs or Arf, alone or in combination, were performed with constant amounts of each cDNA and adding empty-vector DNA (pcDNA3.1) when only one was expressed to keep constant the total amount of DNA. All experiments were performed 48 hours after transfection.

### Immunoblot analysis

Transiently transfected cells with different combinations of PTHR isoforms were lysed with Nonidet P40 (50 mM Tris, 150 mM NaCl, 5 mM EDTA, 0.5% Nonidet P40) supplemented with protease inhibitor mixture I and incubated for 30 minutes on ice. Lysates were centrifuged for 20 minutes at 14,000*g* at 4°C.

Total lysate proteins were analyzed by SDS-PAGE and transferred to Immobilon-P membranes (Millipore, Billerica, MA, USA) using the semidry method (BioRad, Hercules, CA, USA). Nonspecific binding was blocked by incubating the membranes in 5% nonfat milk in Tris-buffered saline plus 0.1% Tween-20 (TBST) for 1 hour at room temperature, followed by overnight incubation with the indicated antibodies (monoclonal anti-Flag and anti-HA antibodies, polyclonal anti-phospho p42/44 and anti-p42/44 antibodies at 1:1000) at 4°C. The membranes then were washed and incubated at room temperature for 1 hour in horseradish peroxidase (HRP)–conjugated goat antirabbit IgG or sheep antimouse IgG diluted 1:2000. Protein bands were visualized with a luminol-based enhanced chemiluminescence substrate.

### Receptor binding

Receptor binding was measured as described previously(19,23) using high-pressure liquid chromatography–purified [^125^I][Nle^8,18^,Tyr^34^]-hPTH(1–34)NH_2_ . Different concentrations of PTH(1–34) or vehicle were added to fresh culture medium bathing confluent cells seeded on 24-well plates. HEK cells were incubated with approximately 100,000 cpm of [^125^I][Nle^8,18^,Tyr^34^]-hPTH(1–34)NH_2_ on ice for 2.5 hours. Nonspecific binding was determined either by parallel incubation of nontransfected cells with [^125^I][Nle^8,18^,Tyr^34^]-hPTH(1–34)NH_2_ or measured in parallel experiments carried out in the presence of 1 µM unlabeled PTH(1–34) and subtracted from total binding to calculate specific binding. After incubation, cells were washed twice with cold PBS and solubilized in 0.2 N NaOH. Cell surface–bound [^125^I][Nle^8,18^,Tyr^34^]-hPTH(1–34)NH_2_ was counted by γ spectrometry. Receptor number (*B*_max_ ) was calculated by nonlinear regression using a homologous binding algorithm (Prism, GraphPad, San Diego, CA, USA).(24)

### Immunofluorescence confocal microscopy

Cells were seeded on coverslips and allowed to settle overnight. Then 100 nM PTH(1–34) was added for the indicated times, and cells were fixed with 4% paraformaldehyde. Permeabilized samples were treated for 10 minutes with 0.1% Triton X-100 in PBS. Nonspecific binding was blocked with 5% goat serum in PBS for 1 hour at room temperature. Polyclonal anti-HA and anti-LAMP-2 and monoclonal anti-Flag or anti-His antibodies were added for 1 hour at room temperature.

After three PBS washes, samples were incubated with Alexa-Fluor 488 or Alexa-Fluor 546 (1:1000) for 1 hour at room temperature. 4',6-Diamidino-2-phenylindole (DAPI) was used to stain the cell nucleus in some samples. Slides were mounted with aqueous mounting medium and examined by confocal microscopy using an Olympus FluoView 1000 (Olympus Corp., Lake Success, NY, USA).

### Receptor internalization

PTHR internalization was measured in cells transiently transfected with HA-PTHR, HA-Δe14-PTHR, or HA-PTHR plus Flag-Δe14-PTHR. Cells were seeded on poly-d-lysine-coated 24-well plates. Confluent cells were treated with PTH and fixed with 3.7% paraformaldehyde at room temperature. After 3 washes with PBS, cells were blocked with 1% bovine serum albumin (BSA) for 45 minutes and incubated with polyclonal anti-HA antibody for 1 hour at room temperature. Cells then were washed with PBS, reblocked with 1% BSA for 15 minutes, and incubated with anti-IgG conjugated with alkaline phosphatase (ELISA protocol) or antirabbit Alexa Fluor 680 nm (flow cytometry protocol) for 1 hour at room temperature. After washing, alkaline phosphatase substrate was added for 30 minutes, 100 µL of the reaction mixture was transferred to a 96-well plate, and absorbance was measured at 405 nm (ELISA protocol).

### Fluorescence resonance energy transfer (FRET)

HEK-293 cells were transiently transfected with the cAMP biosensor EPAC.(25) Cells plated on poly-d-lysine-coated glass 25-mm coverslips were maintained in HEPES/BSA buffer. Coverslips were mounted on the stage of an Olympus IX 71 microscope equipped with a 60× oil-immersion objective and a monochromator (TILL Photonics, Gräfelfing, Germany). FRET was monitored as the emission ratio of YFP and CFP with SlideBook (Intelligent Imaging Innovations, Inc., Denver, CO, USA). FRET was calculated and normalized as described previously.(26) Results are shown as the normalized mean (nFRET) ± SEM.

### Semiquantitative RT-PCR

Total cell RNA was isolated with TRIZOL. Then 400 ng of RNA was reverse-transcribed, and the resulting cDNA was amplified using a commercial kit (Titanium One-Step RT-PCR, Clontech, Palo Alto, CA, USA) with the primers GTCCAGATGCACTATGAG (forward) and GACATTGGTCACACTTGT (reverse), corresponding to nucleotides 1315 to 1332 and 1507 to 1524, respectively, in the human *PTHR* gene (GenBank Accession Number NM 000316). *GAPDH* primers GAGTCAACGGATTGGTCGT (forward) and TTGATTTTGGAGGGATCTCG (reverse) were used for *GAPDH* coamplification as an internal control. PCR products were separated on 2% agarose gels, and bands were visualized by ethidium bromide staining. Quantitative PCR (qPCR) experiments used the same primers. TaqMan MGB probes were obtained by Assay-by-Design (Applied Biosystems). PTHR VIC-TCGCAATCATATACTGTTTCTGCAA-TAMRA and Δe14-PTHR 6FAM-TCAACTCCTTCCAGGTACAAGCTGAGA-TAMRA cDNA was synthesized using AccuScript High Fidelity RT-PCR System (Stratagene, La Jolla, CA, USA) with random hexamer primers, and qPCR was carried out with an ABI PRISM 7500 System (Applied Biosystems) following the manufacturer's instructions.

### Image analysis

Colocalization of Δe14-PTHR within cytoplasmic compartments was analyzed with ImageJ(27) to calculate the Pearson coefficient, which is defined here as the ratio of the covariance of the red and green color images divided by the product of the standard deviation of the normalized image intensities.

### Statistics

Data are presented as the mean ± SE, where *n* indicates the number of independent experiments. Multiple comparisons were evaluated by one- or two-way analysis of variance with posttest repeated measures analyzed by the Bonferroni procedure (Prism, GraphPad). Differences greater than *p* ≤ .05 were assumed to be significant.

## Results

### Expression of Δe14-PTHR in human cells

**Fig. 1:**
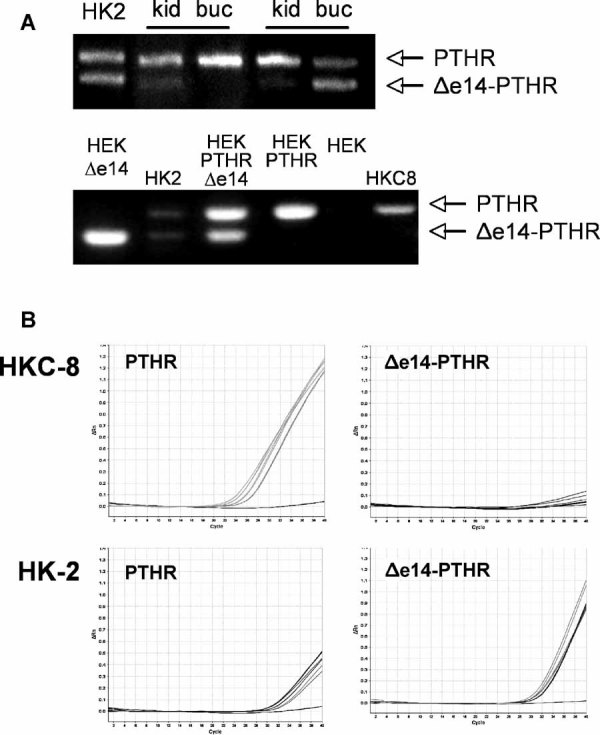
PTHR isoform in human cells. (*A*) mRNA was extracted from renal (kid) and buccal (buc) epithelial cells of two normal subjects or HK2, HKC-8, and HEK-293 cells lines transfected with Δe14-PTHR (e14) and/or PTHR. (*B*) qPCR analysis of mRNA isolated from HKC-8 or HK2 cells. Δ*R_n_* = change in normalized reporter signal. Representative samples of three to five independent determinations are shown. Assays were performed as described in “Materials and Methods.”

Previous data from family B1 GPCRs suggested the possibility of an alternatively spliced form of the PTHR lacking TMD7.(5–8,12) To identify a PTHR isoform with these characteristics in human cells, mRNA from renal tubule cells collected from urine and/or buccal epithelial cells was analyzed. Amplification by RT-PCR generated a fragment of the expected 217 bp indicating *PTHR* gene expression (Fig. 1*A*). Notably, an additional smaller product of 171 bp was detected in renal and in some buccal mRNA samples (Fig. 1*A*), consistent with the size of small *PTHR* transcripts reported in rat kidney cells.(9) The smaller band was sequenced and corresponds to the *PTHR* mRNA with an in-frame 42-bp deletion corresponding to exon 14, which encodes most of TMD7 (data not shown). No mutations were noted in the coding regions or in the corresponding donor and acceptor splice sites.

HK-2 renal tubular epithelial cells expressed both PTHR and Δe14-PTHR forms of the receptor, whereas HKC-8 cells expressed only wild-type PTHR. Full-length and truncated PTH receptors specifically designed were transfected in HEK-293 as a control (Fig. 1*A*, *bottom*). The presence of Δe14-PTHT was corroborated by qPCR using probes specific for this alternatively spliced variant (Fig. 1*B*).

### Δe14-PTHR topology

**Fig. 2:**
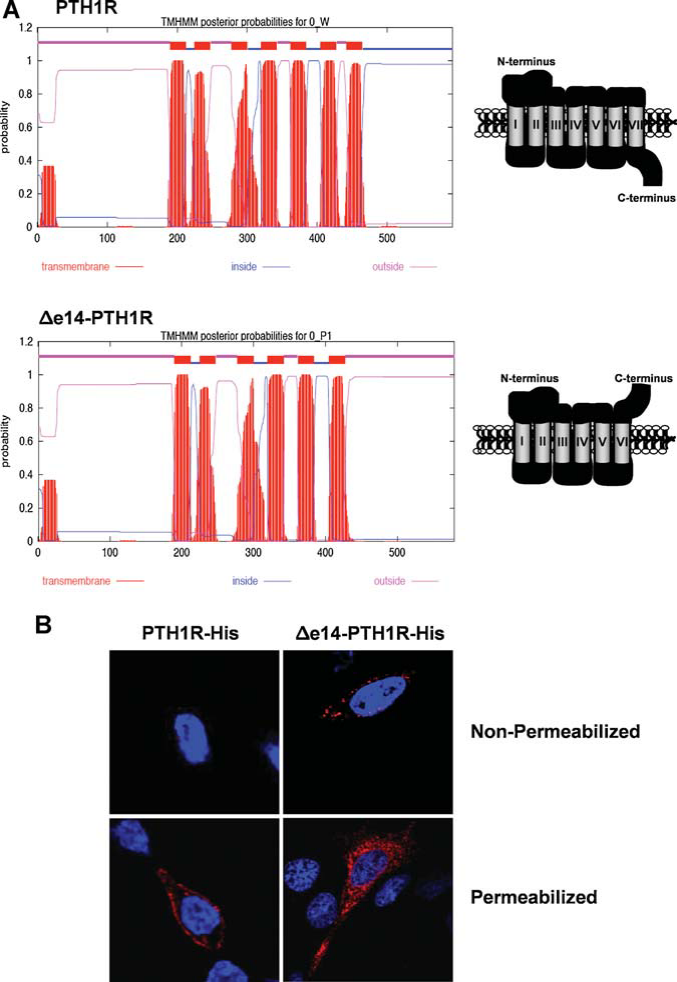
Orientation of Δe14-PTHR C-terminus. (*A*) Prediction of PTHR and Δe14-PTHR topology of transmembrane helices and inverting loop regions. The protein sequences of PTHR and Δe14-PTHR were analyzed with the TMHMM program (http://workbench.sdsc.edu) to predict TMD and intracellular/extracellular loops. Red represents TMD, the intracellular loops are represented in blue, and the extracellular loops are shown in pink. (*B*) Orientation of the C-terminus of PTHR and Δe14-PTHR overexpressed in CHO-N10 cells was assayed by confocal microscopy. Cells transiently transfected with PTHR or Δe14-PTHR labeled at the C-terminus with a polyhistidine tag were either not permeabilized with Triton X-100 (*top panel*) or permeabilized (*bottom panel*) before addition of specific antibody against histidine. DAPI staining was used to identify the nuclei. Similar results were obtained from multiple independent experiments.

We analyzed the predicted topology of the Δe14-PTHR and compared it with the wild-type receptor using the TMHMM algorithm (http://workbench.sdsc.edu), which predicts transmembrane helices and inverted-loop regions based on a hidden Markov model.(28) Whereas the PTHR displayed the expected heptahelical protein conformation with an intracellular C-terminus, the Δe14-PTHR folds with 100% probability as a 6-transmembrane-spanning receptor without TMD7 and with the C-terminus located extracellularly (Fig. 2*A*). To test this prediction, we generated Δe14-PTHR with a polyhistidine (6× His) tag at the C-terminus. The localization of Δe14-PTHR was determined by confocal microscopy with CHO-N10 cells. In nonpermeabilized cells, PTHR was undetectable, consistent with the inaccessible C-terminal epitope tag in the cytoplasm (Fig. 2*B*). Under the same conditions, distinct Δe14-PTHR cell surface fluorescence is present. In permeabilized cells, both Δe14-PTHR and PTHR immunofluorescence are observed (Fig. 2*B*). These findings are compatible with an extracellular localization of the C-terminus of Δe14-PTHR.

### Cytoplasmic Δe14-PTHR expression

**Fig. 3:**
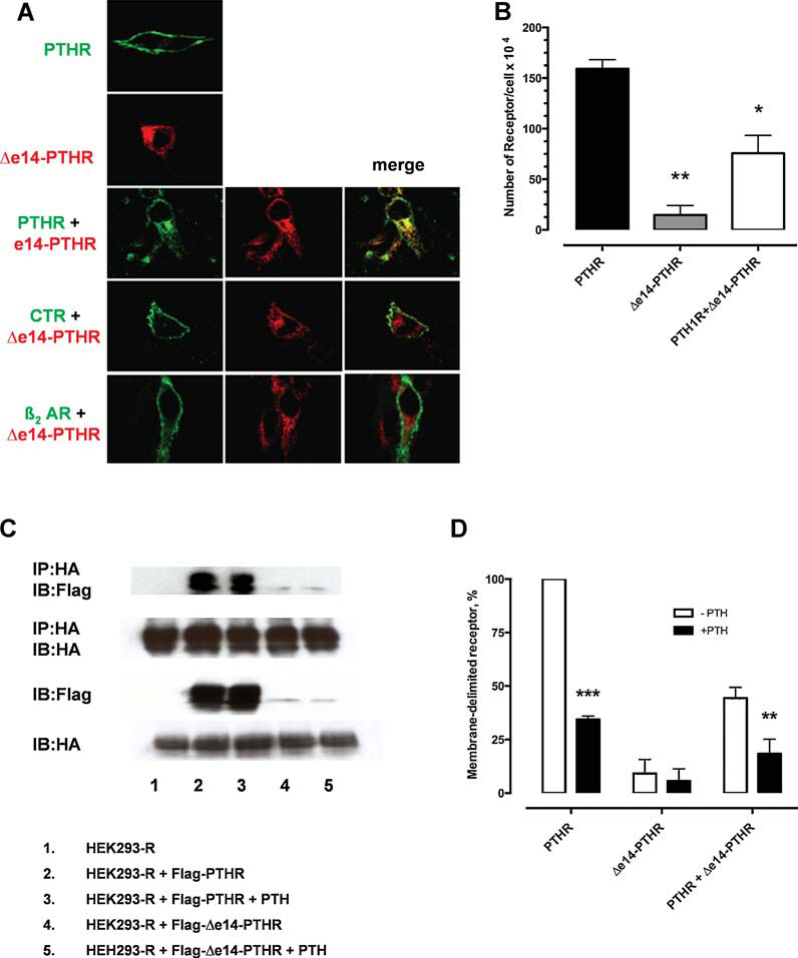
Δe14-PTHR localizes at the cytoplasm and interacts with PTHR. (*A*) HEK-293 cells were transiently transfected with HA-PTHR, HA-calcitonin receptor (CTR), GFP-β_2_ -adrenergic receptor, and/or Flag-Δe14-PTHR, grown on glass cover slips for 48 hours, fixed, and permeabilized as described in “Materials and Methods.” HA-tagged PTHR and CTR were detected using a specific polyclonal primary antibody for HA (1:1000) and Alexa-Fluor 488 (1:2000) (*green*). Flag-tagged Δe14-PTHR was detected using a specific primary antibody for Flag (1:1000) and Alexa-Fluor 546 (1:2000) (*red*). Right panels show the merged images. Colocalization of the green and red labels is shown in yellow. Representative images obtained by confocal microscopy of at least three experiments are illustrated. (*B*) Number of receptors (*B*_max_ ) in HEK-293 cells transiently cotransfected with HA-PTHR, or Flag-Δe14-PTHR and pcDNA3.1, or HA-PTHR and Flag-Δe14-PTHR was calculated as described in “Materials and Methods.” Data are the mean triplicate determinations and are summarized as ± SE of three independent experiments. ^**^p < .01; ^*^p < .05 versus PTHR. (*C*) HEK-293 cells stably transfected with HA-PTHR (HEK-R) were transiently transfected with or without Flag-PTHR or Flag-Δe14-PTHR as indicated. Cells were lysed after 24 hours, and the HA-PTHR/Flag-PTHR and HA-PTHR/Flag-Δe14-PTHR dimers were immunoprecipitated (IP) using the HA.11 monoclonal affinity matrix. Immune complexes were immunoblotted (IB) with anti-HA or anti-Flag antibodies as described in “Materials and Methods.” Total lysates were immunoblotted with anti-HA or anti-Flag antibodies as a transfection control. Representative images of at least three independent experiments are shown. (*D*) HEK-293 cells on 24-well plates were transiently transfected with HA-PTHR, HA-Δe14-PTHR and pcDNA3.1, or HA-PTHR and Flag-Δe14-PTHR. After 48 hours, the cells were incubated in the presence or absence of PTH(1–34) for 30 minutes. Receptor internalization was assayed by ELISA as described in “Materials and Methods.” Similar results were obtained in three independent experiments. ^***^p < .001; ^*^^*^p < .01 versus –PTH.

To assess the subcellular distribution of Δe14-PTHR, we transiently transfected HEK-293 cells with truncated or full-length PTH receptors. Confocal microscopy shows that HA-PTHR clearly localizes to the cell membrane (Fig. 3*A*). Similar results were obtained in CHO-N10 cells and with Flag-PTHR or GFP-PTHR (images not shown). In contrast, Flag-Δe14-PTHR exhibited conspicuously lower cell surface expression but intense cytoplasmic abundance (Fig. 3*A*). HA-Δe14-PTHR also was predominantly cytoplasmic with little plasma membrane expression (image not shown).

We next characterized the influence of Δe14-PTHR on PTHR distribution. Truncated and full-length receptors were cotransfected in HEK-293 and CHO-N10 cells. Whereas PTHR is not normally observed in cytoplasm (Figs. 2*B* and 3), strong cytoplasmic colocalization of GFP-PTHR and Flag-Δe14-PTHR was observed in HEK-293 cells (Fig. 3*A*). Similar results were obtained in CHO-N10 cells (images not shown). These findings suggest that Δe14-PTHR causes retention of PTHR in the cytoplasm.

To determine if the interference by Δe14-PTHR of membrane targeting is specific to the PTHR, we examined the effect of Δe14-PTHR on the localization of the calcitonin receptor (CTR), a family B receptor with a helix 7 isoform, and the β_2_ -adrenergic receptor, a prototype family A receptor. HA-CTR or GFP-β_2_ -adrenergic receptors were cotransfected with Flag-Δe14-PTHR. Both HA-CTR and GFP-β_2_ -adrenergic receptors localized to the plasma membrane and did not colocalize with Δe14-PTHR in the cytoplasm (Fig. 3*A*). When cotransfected with GFP-PTHR, the HA-CTR showed no effects on PTHR expression at the cell membrane (data not shown). Thus the retention of PTHR in the cytoplasm is specific for Δe14-PTHR.

The extent of plasma membrane Δe14-PTHR expression was quantified by ligand-binding experiments. PTHR exhibited 10-fold higher expression than the Δe14-PTHR with 1.60 × 10^6^ PTHRs/cell compared with 0.15 × 10^6^ Δe14-PTHRs/cell (Fig. 3*B* and Supplemental Fig. S1).

In the presence of Δe14-PTHR, cell surface expression of PTHR decreased by 56% (0.7 × 10^6^ receptors/cell). These findings confirm that Δe14-PTHR suppresses PTHR membrane expression. Considering the effects of the TMD7 on PTHR topology, we turned our attention to whether this truncation affects inherent affinity for PTH. Scatchard analysis of ligand-binding showed *K*_*d*_ values of 5 nM for PTHR, 40 nM for Δe14-PTHR, and 12 nM when both receptors were cotransfected.

We performed coimmunoprecipitation experiments to determine directly whether Δe14-PTHR and PTHR interact. Immunoprecipitation of the full-length receptor and immunodetection of PTHR or Δe14-PTHR showed that both receptors homo- or heterodimerize, respectively (Fig. 3*C*). The reverse experiment, where the truncated Δe14-PTHR was immunoprecipitated and the PTHR or Δe14-PTHR was immunoblotted exhibited comparable results (data not shown). In addition to Δe14-PTHR and PTHR heterodimerization, we also observed PTHR homodimerization (Fig. 3*C*). Together these results show that Δe14-PTHR interacts directly with PTHR.

To determine the dynamic behavior of Δe14-PTHR and PTHR and their trafficking response to PTH, we analyzed receptor internalization by an ELISA assay using nonpermeabilized HEK-293 cells. As shown in Fig. 3*D*, the PTHR was efficiently internalized 30 minutes after PTH(1–34). Δe14-PTHR membrane expression was conspicuously lower than that of the PTHR and did not appreciably internalize on PTH stimulation (Fig. 3*D*).

We next examined Δe14-PTHR effects on PTH-induced internalization of the PTHR. Δe14-PTHR decreased PTHR membrane-delimited expression by 52% (Fig. 3*D*). PTH induced proportionately similar PTHR internalization in the presence or absence of Δe14-PTHR (Fig. 3*D*). Similar results were obtained by flow cytometry (data not shown). These findings suggest that Δe14-PTHR does not affect internalization of the reduced subset of membrane-delimited PTHR.

### Retention of Δe14-PTHR in the endoplasmic reticulum

**Fig. 4:**
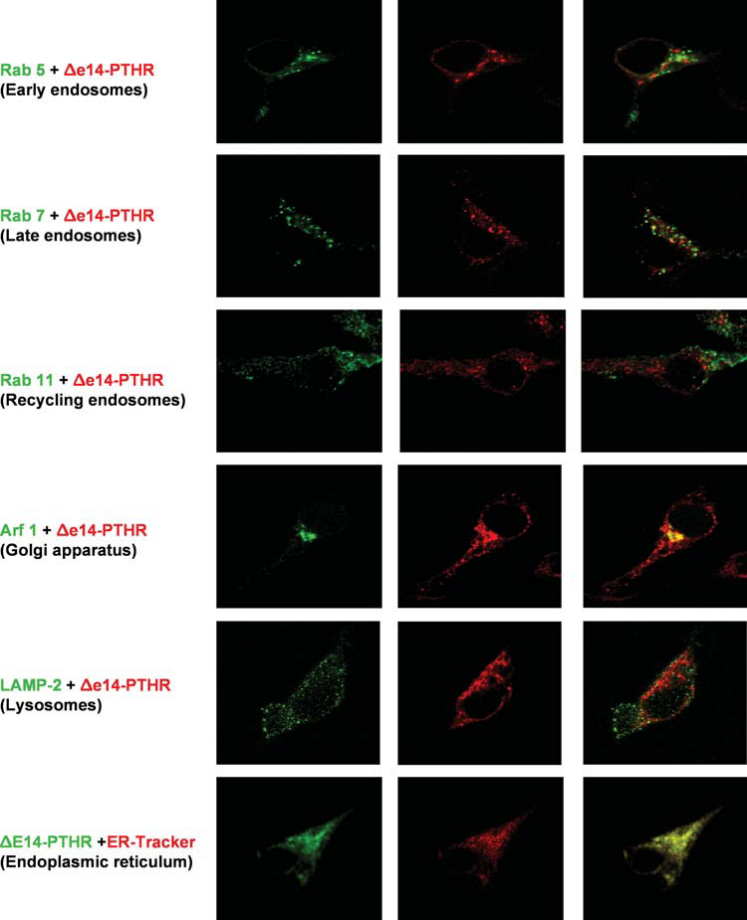
Internalized Δe14-PTHR localizes in the endoplasmic reticulum (ER). HEK-293 cells were transiently cotransfected with Flag-Δe14-PTHR and GFP-Rab 5, GFP-Rab 7, GFP-Rab 11, or GFP-Arf 1 as indicated, grown on glass cover slips for 48 hours, fixed, and permeabilized as described in “Materials and Methods.” Flag-tagged Δe14-PTHR was detected using a specific primary antibody for Flag (1:1000) and Alexa-Fluor 546 (1:2000) (*red*) or Alexa-Fluor 488 (1:2000) (*green*). Lysosomes were detected using a rabbit polyclonal anti-LAMP-2 antibody (1:1000) and Alexa-Fluor 488 (1:2000) (*green*), and the ER was detected using ER-Tracker Red. Right panels show the merged images. Colocalization of the green and red labels is shown in yellow. The cells were examined by confocal microscopy. Representative images of at least three independent experiments are shown.

**Fig. 5:**
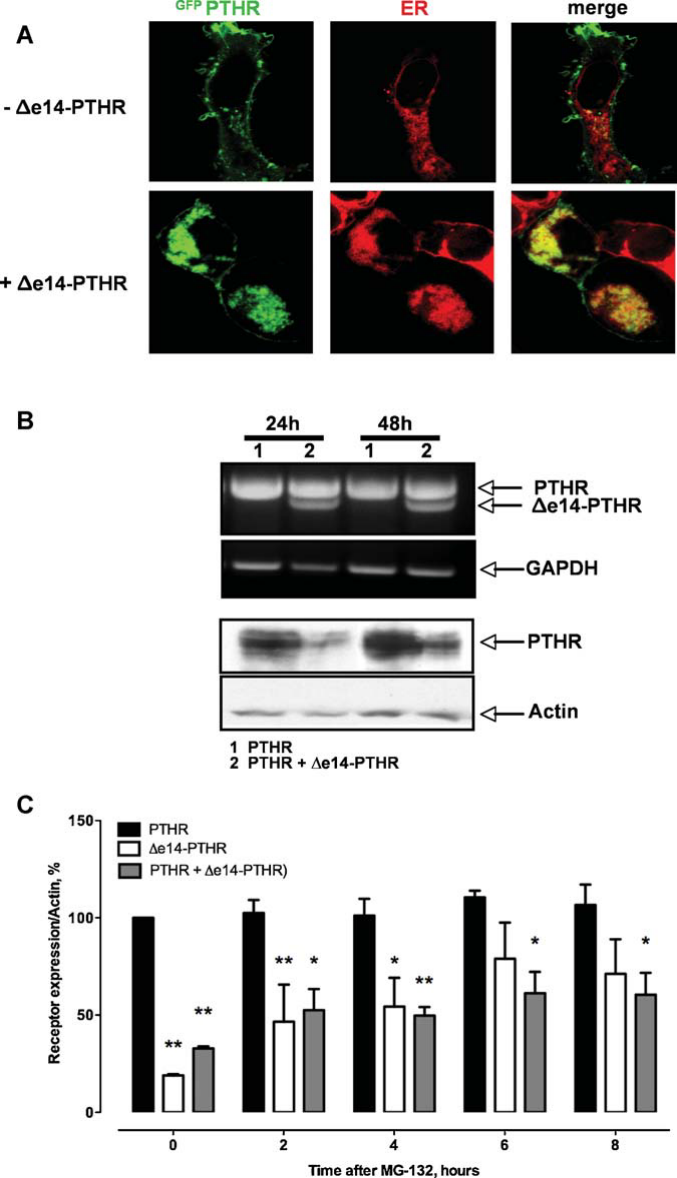
Δe14-PTHR decreases PTHR protein levels. (*A*) HEK-293 cells were transiently transfected with GFP-PTHR ± Flag-Δe14-PTHR as indicated and grown on glass cover slips for 48 hours. ER was detected using ER-Tracker Red. Cells were examined by confocal microscopy. Right panels show the merged images. Colocalization of the green and red labels is shown in yellow. Representative images of at least three independent experiments are shown. (*B*) CHO-N10 cells were transiently cotransfected with HA-PTHR and Flag-Δe14-PTHR or the empty vector pcDNA3.1. After 24 or 48 hours of transfection, mRNA and protein were extracted, and semiquantitative and immunoblot assays were performed as described in “Materials and Methods.” (*C*) HEK-293 cells were transiently transfected with Flag-Δe14-PTHR, HA-PTHR, or Flag-Δe14-PTHR + HA-PTHR (1.5 and 0.5 µg) for 24 hours and treated with the proteasome inhibitor MG-163 for 2 to 8 hours. Total lysates were extracted and immunoblotted as described in “Materials and Methods.” HA and Flag epitopes were detected using specific primary antibodies (1:1000) and HRP-tagged antibodies (1:2000). Data illustrate three or four independent experiments performed in triplicate and were analyzed by two-way ANOVA. ^**^p < .01; ^*^p < .05 versus PTHR.

The difference between PTHR and Δe14-PTHR subcellular localization led us to investigate the intracellular compartmentalization of Δe14-PTHR. We performed confocal microscopy to determine the identity of endosomes containing Δe14-PTHR in HEK-293 cells transfected with either green fluorescent protein (GFP)–tagged Rab5, -7, or -11 or Arf 1, GTPases that control trafficking of early and late, recycling, and Golgi network endosomes, respectively. Modest levels of Δe14-PTHR were found in Rab11^+^ and Arf1^+^ compartments, corresponding to pericentriolar recycling endosomes and the trans-Golgi network (Fig. 4 and Table 1). No significant localization of Δe14-PTHR was observed with Rab5^+^ or -7^+^ early and late endosomes, respectively (Fig. 4 and Table 1). To determine if Δe14-PTHR is targeted to the endocytic degradative pathway or endoplasmic reticulum (ER), we used a lysosomal-associated membrane protein (LAMP-2) antibody or a fluorescent ER-Tracker, respectively, in HEK-293 cells transfected with HA-Δe14-PTHR. Although Δe14-PTHR was not found in LAMP-2^+^ lysosomes, extensive Δe-14PTHR was observed within ER (Fig. 4 and Table 1). These results, along with the previous findings showing limited Δe14-PTHR expression at the cell surface, suggest an early impairment of Δe14-PTHR trafficking to the membrane and retention within the ER. In contrast, PTHR is not detectable in Rab5, -7, or -11, Arf 1, LAMP-2-positive compartments or in ER (Supplemental Fig. S2). Thus, under resting conditions, the PTHR is found only at the cell membrane. However, in the presence of Δe14-PTHR, considerable ER accumulation of PTHR is observed (Fig. 5*A*).

**Table 1:** Cytoplasmic Δe14-PTHR Accumulates in Endoplasmic Reticulum (ER)

	Arf 1	Rab 5	Rab 7	Rab 11	LAMP-2	ER
	
	*r*, %					
PTHR	0.27 ± 0.07	0.39 ± 0.07	0.36 ± 0.06	0.55 ± 0.05	0.29 ± 0.02	0.16 ± 0.03
Δe14-PTHR	0.55 ± 0.16	0.29 ± 0.27	0.34 ± 0.16	0.55 ± 0.17	0.25 ± 0.09	0.70 ± 0.08*

*Note:* The Pearson correlation coefficient *r* was calculated with ImageJ.(27) The calculation shows colocalization of Δe14-PTHR with Arf 1 (Golgi apparatus), Rab 5 (early endosomes), Rab 7 (late endosomes), Rab 11 (recycling endosomes), LAMP-2 (lysosomes), and endoplasmic reticulum (ER). Technical details are described in “Materials and Methods.”

* *p* < .5 significant positive colocalization. *n* = 5 to 8 independent observations for each condition.

### Δe14-PTHR decreases PTHR protein expression

Decreased cell membrane Δe14-PTHR expression combined with cytoplasmic accumulation raised the possibility that these effects could be due to decreased protein synthesis alone or in combination with increased receptor degradation. Indeed, we observed decreased Δe14-PTHR protein expression levels compared with PTHR (Fig. 5*B*). Moreover, cotransfection of Δe14-PTHR impaired PTHR expression (Fig. 5*B*). Notably, no differences in *PTHR* mRNA expression were observed in cells cotransfected with Δe14-PTHR (Fig. 5*B*). Similar data were obtained in HEK-293 and COS-7 cells and by PCR (data not shown).

Net receptor protein expression is a balance between synthesis and degradation. To test the hypothesis that proteasome- or lysosome-dependent degradative mechanisms contribute to diminished Δe14-PTHR protein levels, HEK-293 cells transfected with Δe14-PTHR were treated with MG-132 or chloroquine, proteasome and lysosome inhibitors, respectively. Δe14-PTHR protein expression rebounded after proteasome blockade (*t*_1/2_ = 5.39 hours; Fig. 5*C*). Lysosome inhibition did not affect Δe14-PTHR degradation (data not shown). Within experimental error, neither proteosomal nor lysosomal degradation of PTHR was detected (data not shown). Thus Δe14-PTHR is metabolized by ubiquitination and targeted to proteasomes. When Δe14-PTHR was cotransfected with PTHR, however, PTHR protein levels that were diminished in the presence of Δe14-PTHR now increased toward basal expression values when pretreated with the proteasome inhibitor (*t*_1/2_ = 2.0 hours; Fig. 5*C*). Again, lysosomal inhibition was without effect (data not shown).

### Δe14-PTHR inhibits PTHR signaling

**Fig. 6:**
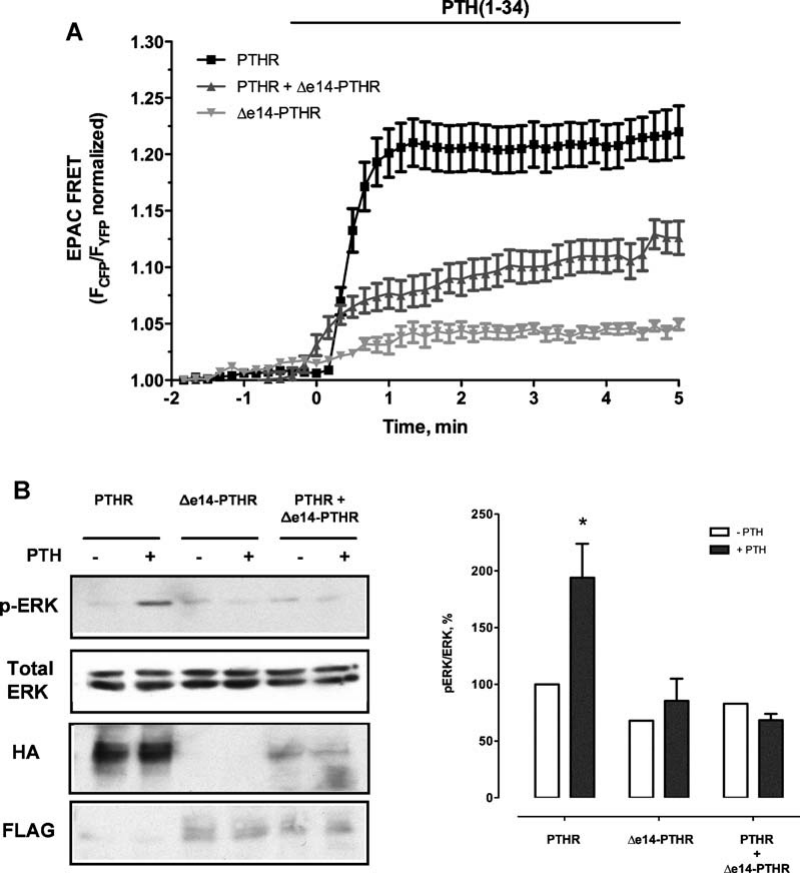
Δe14-PTHR impaired PTHR-induced cAMP activation and ERK phosphorylation triggered by PTH(1–34). (*A*) HEK-293 cells transiently transfected with HA-PTHR, EPAC and pcDNA3.1, and/or Flag-Δe14-PTHR were treated with 100 nM PTH(1–34) for 5 minutes. cAMP accumulation was measured by FRET, as described in “Materials and Methods.” Data are the average of triplicate independent determinations. (*B*) CHO-N10 cells transiently transfected with different combinations of HA-PTHR, pcDNA3.1, and/or Flag-Δe14-PTHR as indicated were grown on 6-well plates for 48 hours and serum-starved for 2 hours before stimulation with 100 nM PTH(1–34) for 10 minutes. Total lysates were extracted, and immunoblotting was performed as described in “Materials and Methods.” Phospho-p44/42, total p44/42, and HA and Flag epitopes were detected using specific primary antibodies (1:1000) and HRP-tagged antibodies (1:2000). Upper panels show representative immunoblot images. Data illustrate three independent experiments performed in triplicate. ^*^p < .05 versus control.

As shown earlier, the absence of TMD7 impairs membrane localization of Δe14-PTHR and alters its subcellular distribution, suggesting that its biologic response to PTH likely would be compromised. We therefore characterized the signaling capability of Δe14-PTHR by measuring cAMP and ERK responses to PTH, two well-established and independent signaling mechanisms. Using the cAMP FRET biosensor EPAC (exchange protein directly activated by cAMP), we observed a rapid increase of cAMP formation (denoted as the CFP/YFP ratio) triggered by PTH(1–34) in HEK-293 cells transfected with PTHR (*t*_1/2_ = 0.42 ± 0.05 minutes; Fig. 6*A*). The longer *t*_1/2_ of 0.86 ± 0.16 minutes for the Δe14-PTHR suggests that cAMP signaling is impaired (Fig. 6*A*). Additionally, we observed limited ERK phosphorylation in response to PTH(1–34) in CHO-N10 cells transfected with Δe14-PTHR compared with PTHR (Fig. 6*B*).

Because Δe14-PTHR affects PTHR membrane expression and subcellular distribution, we predicted that the truncated receptor also disrupts PTHR signaling. To test this idea, we measured cAMP activation and ERK phosphorylation in HEK-293 cells transiently transfected with HA-PTHR with or without Flag-Δe14-PTHR. Cotransfection of PTHR with Flag-Δe14-PTHR strongly inhibited PTH(1–34)-triggered cAMP formation, as determined by FRET (Δe14-PTHR + PTHR *t*_1/2_ = 0.65 ± 0.08 minutes versus PTHR *t*_1/2_ 0.42 ± 0.05 minutes; Fig. 6*A*). The inhibitory action of Δe14-PTHR was specific in that Δe14-PTHR did not interfere with norepinephrine-stimulated cAMP formation by the β_2_ -adrenergic receptor (*t*_1/2_ = 1.157 ± 0.008; *t*_1/2_ = β_2_ -adrenergic receptor + Δe14-PTHR = 1.190 ± 0.015, NS). Furthermore, cotransfection of Δe14-PTHR with PTHR abolished PTH-induced ERK phosphorylation (Fig. 6*B*).

## Discussion

This study reveals the presence of a novel, alternatively spliced PTHR isoform in renal tubular epithelial cells and characterizes its trafficking and signaling, as well as its structural and functional interactions with the full-length PTHR. The low abundance of Δe14-PTHR at the plasma membrane underscores the importance of the TMD7 for proper receptor targeting and integration at the cell surface and for membrane retention. The structural basis for the critical role of this domain for accurate membrane receptor localization is not well understood. Failure of receptor export or decreased stability at the membrane could account for reduced Δe14-PTHR cell surface expression. A GFF motif within the conserved region of TMD7 is indispensable for CRHR membrane expression.(8) This motif, which also is present in the PTHR, may be essential to form the seventh hydrophobic helix, and in its absence, the consequent protein misfolding does not allow the receptor to be transported through the endoplasmic reticulum (ER).(8) Other checkpoint motifs described for vasopressin V_2_ , angiotensin II, dopamine D_1_ , V1b/V3, and β_2_ -adrenergic receptors are necessary for ER-to-Golgi transfer.(29–33) However, these motifs are absent in the PTHR C-terminus. Alternatively, excision of the Δe14-PTHR TMD7 could generate a motif that inhibits transit of the truncated receptor to the membrane by unmasking a cryptic retention signal, as observed in γ-aminobutyric acid (GABA) receptors.(34)

Dimerization is required for some GPCRs to be transported to the plasma membrane.(35) The C-terminus of the GABA_B_ receptor, for instance, is critical to promote receptor dimerization. More specifically, heterodimerization of GABA_B_ receptors uses the C-terminal retention motif RXR(R),(36) which also is present in the PTHR. It is thus possible that the nascent PTHR is formed as a dimer that dissociates in the ER before transport to the plasma membrane. Recent evidence demonstrates that the PTHR is targeted to the plasma membrane as a dimer and dissociates on binding PTH.(37) PTHR–Δe14-PTHR heterodimers may not be able to dissociate, accounting for the cytoplasmic accumulation of PTHR in the presence of Δe14-PTHR. Heterodimerization of CTR with its truncated isoform, a process that involves the C-terminus, prevents transport of the receptor to the cell surface.(5) The aberrant orientation of the Δe14-PTHR C-terminus and protein misfolding could act on the PTHR in a similar manner, causing accumulation in the ER and retention of the full-length PTHR, thereby impairing its transport to the cell membrane.

In addition to lower expression at the cell surface, Δe14-PTHR exhibits lower affinity for PTH. Thus TMD7 influences PTH binding, as it does calcitonin binding to CTR,(5) although TMD7 is not necessary for agonist binding to CRH-R1d.(6) Hence similar motifs are capable of exerting distinct roles on ligand affinity to family B GPCRs. Compared with their full-length receptor counterparts, CRHR and CTR isoforms lacking the seventh TMD exhibited impaired ligand-stimulated cAMP formation(6,38) or limited coupling to Gs, Gq, Gi, and Go in the case of the CRHR isoform, CRH-R1d.(6) The fact that the *t*_1/2_ for adenylyl cyclase activation by PTH was reduced suggests that Δe14-PTHR coupling to adenylyl cyclase is compromised. This kinetic manifestation arises as a consequence of decreased activated (receptor-ligand) complex. By contrast, normalizing the extent of cAMP formation to receptor number indicates that there is no change in Δe14-PTHR intrinsic activity (ie, the magnitude of the response). Similar observations were reported for the truncated isoform of CTR, which failed to mobilize intracellular calcium or phosphorylate ERK.(8) Thus the reduced signaling by Δe14-PTHR is likely due to a combination of the 10-fold lower expression of Δe14-PTHR at the cell membrane and diminished ligand affinity.

Several key signaling motifs situated within the PTHR intracellular tail are inaccessible in the Δe14-PTHR owing to its extracellular location. This also could contribute importantly to the diminished signaling by the Δe14-PTHR. For instance, mutations in the juxtamembrane region of the C-tail between amino acids 468 and 491 of the PTHR disrupt Gßγ interactions with the receptor, block PTH signaling by phospholipase C and ERK, and markedly reduce cAMP signaling.(39) Furthermore, the PTHR C-terminus contains several proline-rich motifs that are essential to trigger ERK phosphorylation by c-Src and arrestin activation(36) that would not be available in Δe14-PTHR. Negative and positive regulators of PTHR endocytosis that are present within the upstream region of the PTHR intracellular tail(40) would no longer exert their actions in the Δe14-PTHR. Finally, cytoplasmic PDZ scaffolding proteins such as NHERF1 that interact with the C-terminus and regulate signaling and PTHR trafficking(19,23,41–43) would be incapable of exerting their modulatory actions on the Δe14-PTHR. Thus the redirected extracellular C-terminus of the Δe14-PTHR, in combination with limited Δe14-PTHR expression at the plasma membrane also may contribute to the reduced signaling of this naturally occurring receptor isoform.

Protein synthesis is regulated at multiple levels during transcription and translation. Our results show that diminished PTHR expression is not due to downregulation at transcriptional levels because similar *PTHR* mRNA expression was observed in the presence or absence of Δe14-PTHR. This suggests possible posttranscriptional modulation of PTHR expression by the truncated receptor. Proteins localized at the plasma membrane usually are degraded by lysosomes,(44) whereas misfolded proteins that accumulate in cytoplasmic compartments such as the ER, the ER/Golgi intermediate compartment (ERGIC), or the Golgi apparatus eventually are targeted for metabolism by the ubiquitination- and proteasome-dependent ER-associated degradation pathway (ERAD) or by mechanisms that remain unknown, respectively.(45,46) Δe14-PTHR could interact with PTHR in the ER, ERGIC, or Golgi compartments, leading to its retention and subsequent proteolysis by proteasome degradation. The response to PTH, as in HK-2 cells, could be diminished owing to expression of the Δe14-PTHR compared with other cells that do not express this isoform.

Exon skipping is a common mechanism of genomic combinatorial control of alternative splicing.(47) The introns flanking the skipped exon typically possess specific sequences, in addition to the canonical splice donor and acceptor sequences that regulate where skipping occurs. A G-rich region distal to the 5' splice donor and a C-rich region proximal to the 3' splice acceptor play key roles in this process.(48) These regions form a stem-loop structure in the heteronuclear RNA (hnRNA) that makes it possible to bring, in the case of the PTHR, exons 13 and 15 close together and permit the deletion of exon 14. The small, 42-bp size of exon 14 makes it an ideal candidate for exon skipping. In a stretch of 11 bases, 8 are complementary. Moreover, although there is significant complementarity between the 5' G-rich region upstream of the exon 14 and the C-rich region downstream of exon 14, it is not perfect. This could permit small nuclear ribonuclear proteins (snRNPs) that regulate the splicing process to promote inclusion or exclusion of exon 14.

Pseudohypoparathyroidism type 1b (PHP1b) is characterized by renal PTH resistance accompanied by hypocalcemia, hyperphosphatemia, and elevated serum PTH levels.(49) Defective genomic imprinting of GNAS accounts for most cases of familial PHP1b. However, autosomal dominant inheritance does not explain the majority of cases of PHP1b(50) or a significant portion of PHP1a.(51) Regulated expression of Δe14-PTHR by snRNPs might affect PTHR abundance in the kidney. Accumulation of Δe14-PTHR in cells expressing PTHR from different tissues, we propose, inhibits signaling and function of the full-length receptor and could explain PTH resistance in some cases of pseudohypoparathyroidism and perhaps in other forms of PTH or PTH-related protein (PTHrP) resistance of unknown origin.

In summary, Δe14-PTHR is present in renal tubular epithelial cells, where it exhibits reduced anchorage to the plasma membrane, mislocation of its C-terminus to the extracellular compartment, and accumulation in the ER and displays impaired cAMP and ERK signaling. Moreover, Δe14-PTHR decreases PTHR cell surface expression and protein levels, forms heterodimers with PTHR, and also inhibits PTHR-mediated cAMP and ERK signaling. Exon 14 deletion may arise from a regulated but as yet poorly understood pattern of hnRNA complementarity common to family B receptors.
