# Supplementary material for: A Naturally Occurring Isoform Inhibits Parathyroid Hormone Receptor Trafficking and Signaling
Source: J Bone Miner Res. 2010 Jun 24;26(1):143–55. doi: 10.1002/jbmr.167 (PMC3179322; doi:10.1002/jbmr.167)
Supplement: Supplementary file 1 [file jbmr0026-0143-SD1.doc]

**
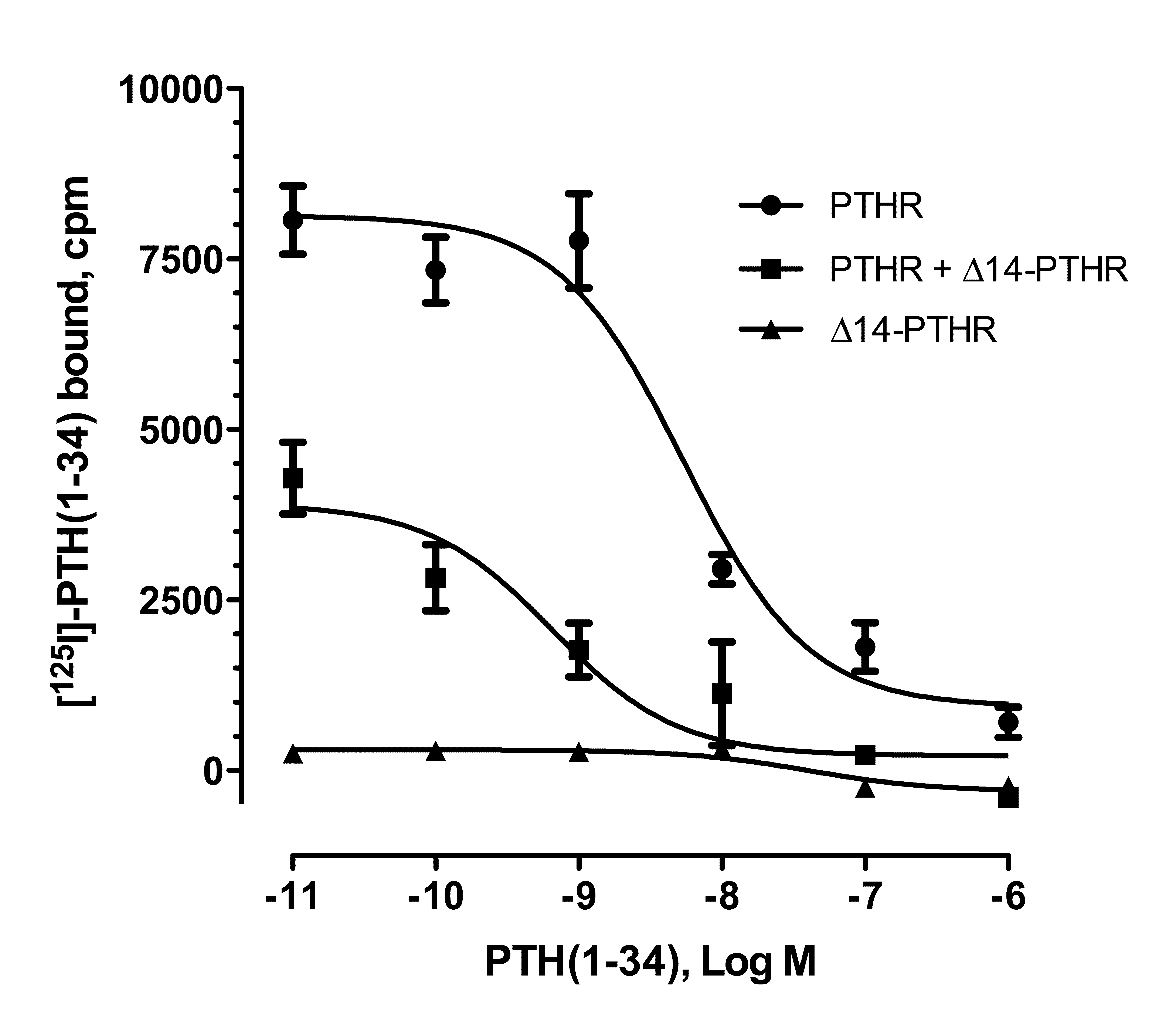
**

**Supplementary Fig. 1.** Cell surface binding of [^125^I]PTH(1-34) in HEK-293 cells transiently transfected with PTH1R or ∆e14-PTH1R and pcDNA3.1, or cotransfected with PTH1R and ∆e14-PTH1R. Maximum specific binding (Bmax) was calculated as described in Materials and Methods using Prism. Bmax = 7201 for PTHR; 3688 for PTHR+∆e14-PTHR, and 611 for ∆e14-PTHR. Data represent the mean ± SE of triplicate determinations.

**
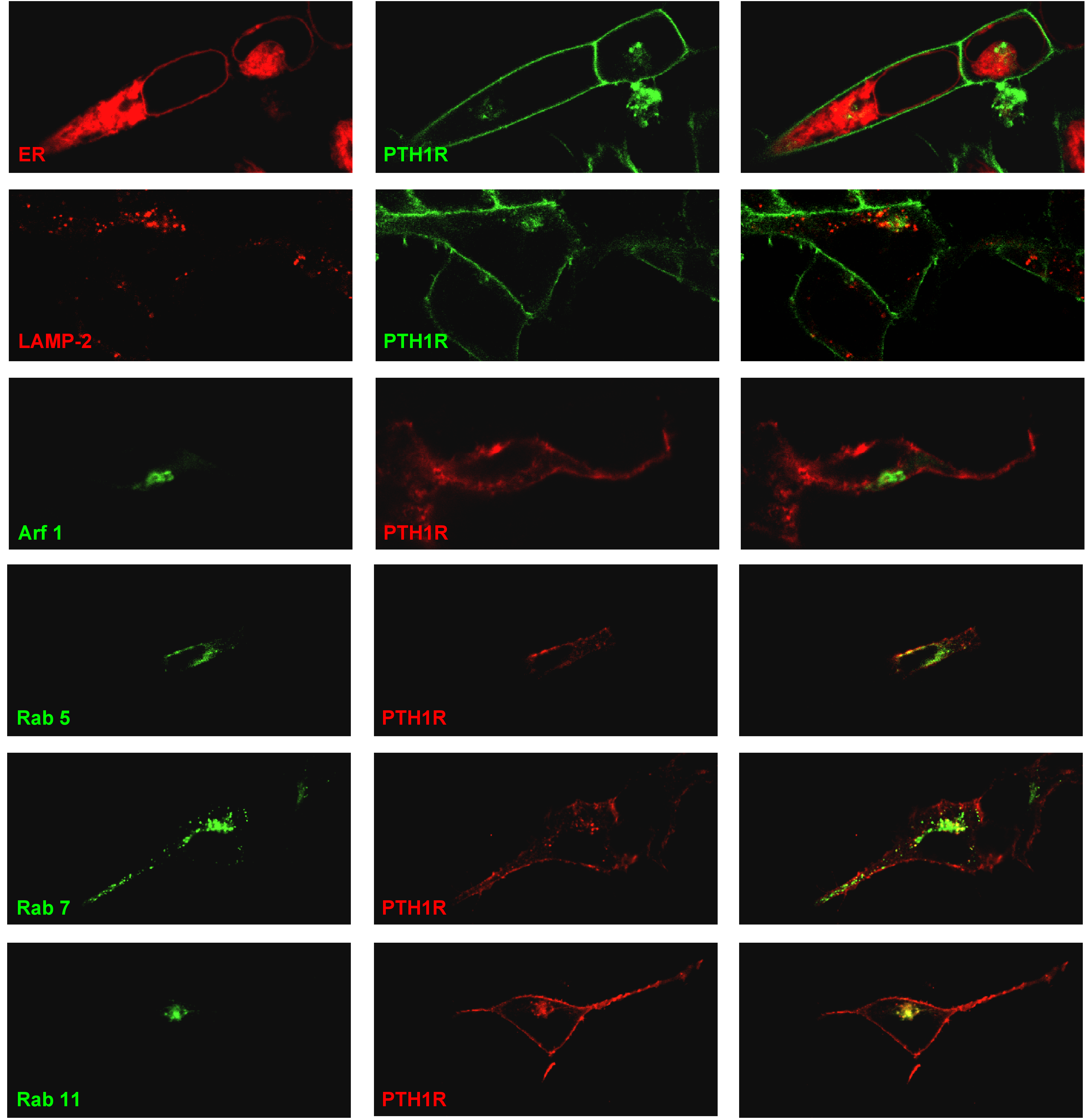
**

**Supplementary Fig. 2.** PTHR localizes at the cytoplasmic membrane. HEK-293 cells were transiently co-transfected with Flag-PTHR or GFP-PTHR and GFP-Rab 5, GFP-Rab 7, GFP-Rab 11, or GFP-Arf 1 as indicated, grown on glass cover slips for 48 hours, fixed and permeabilized as described in Materials and Methods. Flag tagged PTHR was detected using a specific primary antibody for Flag (1:1000) and Alexa-Fluor 546 (1:2000) (red). In cells transfected with GFP-PTHR lysosomes were detected using a rabbit monoclonal anti-LAMP-2 antibody (1:1000) and Alexa-Fluor 546 (1:2000) (red) and endoplasmic reticulum was detected using ER-Tracker™ Red. Right panels show the merged images. Colocalization of the green and red labels are shown in yellow. The cells were examined by confocal microscopy. Representative images of at least 3 independent experiments are shown.
